# Heme metabolism genes Downregulated in COPD Cachexia

**DOI:** 10.1186/s12931-020-01336-w

**Published:** 2020-05-01

**Authors:** Ava C. Wilson, Preeti L. Kumar, Sool Lee, Margaret M. Parker, Itika Arora, Jarrett D. Morrow, Emiel F. M. Wouters, Richard Casaburi, Stephen I. Rennard, David A. Lomas, Alvar Agusti, Ruth Tal-Singer, Mark T. Dransfield, J. Michael Wells, Surya P. Bhatt, George Washko, Victor J. Thannickal, Hemant K. Tiwari, Craig P. Hersh, Peter J. Castaldi, Edwin K. Silverman, Merry-Lynn N. McDonald

**Affiliations:** 1 Department of Epidemiology, School of Public Health, University of Alabama at Birmingham, Birmingham, AL USA; 2 Division of Pulmonary, Allergy and Critical Care Medicine, Department of Medicine, University of Alabama at Birmingham, Birmingham, AL USA; 3 Channing Division of Network Medicine, Brigham and Women’s Hospital, Boston, MA USA; 4 Centre of expertise for chronic organ failure, Horn, the Netherlands; 5 Rehabilitation Clinical Trials Center, Los Angeles Biomedical Research Institute at Harbor Harbor-UCLA Medical Center, Torrance, CA USA; 6 Department of Medicine, Nebraska Medical Center, Omaha, NE USA; 7 BioPharmaceuticals R&D, AstraZeneca, Cambridge, UK; 8 UCL Respiratory, Division of Medicine, University College London, London, UK; 9 Fundació Investigació Sanitària Illes Balears (FISIB), Ciber Enfermedades Respiratorias (CIBERES), Barcelona, Catalunya Spain; 10 Thorax Institute, Hospital Clinic, IDIBAPS, University of Barcelona, Barcelona, Spain; 11 GSK R&D, Collegeville, PA USA; 12 Division of Pulmonary and Critical Care Medicine, Brigham and Women’s Hospital, Boston, MA USA; 13 Department of Biostatistics, School of Public Health, University of Alabama at Birmingham, Birmingham, AL USA; 14 Department of Genetics, University of Alabama at Birmingham, Birmingham, AL USA

**Keywords:** Chronic obstructive pulmonary disease

## Abstract

**Introduction:**

Cachexia contributes to increased mortality and reduced quality of life in Chronic Obstructive Pulmonary Disease (COPD) and may be associated with underlying gene expression changes. Our goal was to identify differential gene expression signatures associated with COPD cachexia in current and former smokers.

**Methods:**

We analyzed whole-blood gene expression data from participants with COPD in a discovery cohort (COPDGene, *N* = 400) and assessed replication (ECLIPSE, *N* = 114). To approximate the consensus definition using available criteria, cachexia was defined as weight-loss > 5% in the past 12 months or low body mass index (BMI) (< 20 kg/m^2^) and 1/3 criteria: decreased muscle strength (six-minute walk distance < 350 m), anemia (hemoglobin < 12 g/dl), and low fat-free mass index (FFMI) (< 15 kg/m^2^ among women and < 17 kg/m^2^ among men) in COPDGene. In ECLIPSE***,*** cachexia was defined as weight-loss > 5% in the past 12 months or low BMI and 3/5 criteria: decreased muscle strength, anorexia, abnormal biochemistry (anemia or high c-reactive protein (> 5 mg/l)), fatigue, and low FFMI. Differential gene expression was assessed between cachectic and non-cachectic subjects, adjusting for age, sex, white blood cell counts, and technical covariates. Gene set enrichment analysis was performed using MSigDB.

**Results:**

The prevalence of COPD cachexia was 13.7% in COPDGene and 7.9% in ECLIPSE*.* Fourteen genes were differentially downregulated in cachectic versus non-cachectic COPD patients in COPDGene (FDR < 0.05) and ECLIPSE (FDR < 0.05).

**Discussion:**

Several replicated genes regulating heme metabolism were downregulated among participants with COPD cachexia. Impaired heme biosynthesis may contribute to cachexia development through free-iron buildup and oxidative tissue damage.

## Background

Chronic Obstructive Pulmonary Disease (COPD) is a leading causes of death worldwide, and it maintains increasing morbidity and mortality [1]. COPD is diagnosed based on reduced, irreversible lung function however, comorbidities are common with COPD and negatively impact survival [2]. Muscle wasting and cachexia are relevant comorbidities among COPD patients with prevalence increasing with stage of illness [2]. Cachexia in COPD may be associated with underlying changes in gene expression that could provide valuable insights for surveillance and drug development.

Collectively, there have been limited investigations examining differential gene expression in the context of COPD cachexia. Previous studies of gene expression changes in COPD cachexia have had small sample sizes, non-disease controls for comparison, employed a candidate-gene approach, or have focused on proxies for cachexia such as low fat-free mass index (FFMI) [3, 4]. Nonetheless, these studies have highlighted interesting genes with relevant biology to cachexia such as *FOXO1* and *FOXO3* [5]. The candidate-gene expression approach is limited to our understanding of known biology. In contrast, the genome-wide method is an agnostic, hypothesis-free approach, that has elucidated novel molecular mechanisms of COPD pathogenesis [6]. A challenge for implementing the genome-wide approach is that a large number of study participants are typically required to achieve a genome-wide level of significance. Although low FFMI and low BMI are components of the multifactorial definition of cachexia, these measures alone are not sufficient to monitor cachexia in the entire population at risk [7].

Our goal was to identify and explore differentially expressed genes associated with COPD cachexia. To achieve our objective, we utilized genome-wide expression data to conduct differential gene expression analyses aimed at deciphering the molecular mechanisms associated with cachexia in COPD patients from COPDGene [8]. We then tested whether significantly differentially expressed genes would be replicated in a second cohort of COPD patients from the ECLIPSE study [9].

## Methods

### Study design and ethics

Institutional Review Board approval for all analyses was obtained from the University of Alabama at Birmingham and have been performed in accordance with the ethical standards laid down in the 1964 Declaration of Helsinki and its later amendments. All subjects included in this analysis provided written informed consent prior to their inclusion in the respective studies mentioned below. All statistical analyses were performed in R (version 3.5.3).

### Study populations

For both cohorts, COPD was defined as airflow obstruction using post-bronchodilator spirometry as GOLD grade 2 (FEV1/FVC < 0.7 and 50% < FEV1 < 80% predicted; FEV1: forced expiratory volume in one second, FEV1/FVC: FEV1 expressed as a percentage of forced vital capacity) or higher [10]. Discovery analyses were performed in 400 participants with COPD from the COPDGene study (NCT00608764; www.copdgene.org). COPDGene is a multicenter, observational study intent on characterizing progression of and genetic susceptibility to COPD, the details of which have been described previously [8]. In brief, COPDGene enrolled Non-Hispanic White and African American current or former smokers, with at least a 10 pack-year smoking history, aged 45 to 80 years to follow longitudinally, with two visits 5 years apart. RNA samples were collected at the five-year follow-up visit. Replication analyses were performed in 114 participants with GOLD 2–4 COPD enrolled in the ECLIPSE (Evaluation of COPD Longitudinally to Identify Predictive Surrogate Endpoints; SCO104960, NCT00292552; www.eclipse-copd.com) cohort. In ECLIPSE, phenotype data was obtained via the database of Genotypes and Phenotypes (dbGaP accession: phs001252.v1.p1) and gene expression data from the Gene Expression Omnibus (GEO: GSE76705). In brief, ECLIPSE [9] was a three-year observational study of subjects with COPD aged 45 to 75 years with a smoking history of ten or more pack-years. Like COPDGene, smaller numbers of control subjects were enrolled in ECLIPSE, who were not included in analysis. Forty-six centers across twelve countries collected data at baseline, 3 months, 6 months, and then every 6 months for three years. The main goal of ECLIPSE was to identify biomarkers associated with the progression of disease severity.

### Phenotype data

In COPDGene, cachexia was defined at visit two, approximately five years from baseline, as either self-reported, unintentional weight loss greater than 5% in the past year or low BMI (< 20 kg/m^2^) in addition to one of three criteria: low 6MWD (< 350 m; a surrogate for decreased muscle strength), anemia (hemoglobin< 12 g/dl) or low FFMI (FFMI < 15 kg/m^2^ among women and FFMI < 17 kg/m^2^ among men). Low 6MWD and anemia were classified using visit two data. FFMI was derived from an algorithm incorporating pectoralis muscle area on baseline chest computed tomography scans [11].

In ECLIPSE***,*** cachexia was defined according to the Evans consensus definition [12] at year 1 of the study as: weight loss greater than 5 % in the past 12 months or low BMI (< 20 kg/m^2^) and at least three of the five criteria: low six-minute walking distance (a surrogate for decreased muscle strength), anorexia, abnormal biochemistry (anemia or high CRP), fatigue, and low FFMI [12]. FFM was measured using bioelectrical impedance. Low FFMI was defined as < 15 kg/m^2^ among women and < 17 kg/m^2^ among men. Abnormal biochemistry was defined as having anemia (hemoglobin< 12 g/dl) or high CRP (> 5 mg/l). Six-minute walking distance (6MWD) < 350 m [13] was used to classify decreased muscle strength [13]. Anorexia and fatigue were only available at baseline and end of study. Anorexia was classified if a participant reported not feeling like eating for 3 or more days in the week prior to either the baseline or 3-year follow-up visit. Fatigue was defined at either baseline or 3-year visits as a FACIT (Function Assessment of Chronic Illness Therapy) questionnaire score less than 34 [14].

### Gene expression data

In COPDGene, RNA-sequencing was performed using whole-blood samples collected at visit 2 (~Year 5) from 400 current or former smokers with COPD. RNA-sequencing alignment and quality control was previously describe in detail [6]. Transcripts were included in analyses if expressed at levels > 1 count per million mapped reads in > 10 subjects. Potential batch effects and unknown artifacts were removed using the SVAseq R package [15] to estimate surrogate variables (SV’s) while accounting for the presence of known covariates: age, sex, library construction batch, and white blood cell (WBC) count percentages. Principle component analysis (PCA) was performed on SV residualized expression data to ensure minimal systematic bias (Supplementary Figure 1).

In ECLIPSE, normalized gene expression data was obtained from GEO (GEO: GSE76705). Sample preparation and quality control for gene expression data obtained from ECLIPSE have been previously described [16, 17]. Briefly, gene expression in ECLIPSE was obtained from whole-blood samples collected at baseline and profiled using the Affymetrix Human U133 Plus2 array. Gene expression data were log-transformed, normalized, then background corrected through multi-array averaging and quantile normalization using the affy Bioconductor package [18]. Probes with low variance (< 50%) and those which did not annotate within a specific gene were removed, leaving a dataset of 21,156 probes for analysis in 114 COPD cases. The amount of missing data for each probe was assessed for quality control, and no probes were removed due to missingness, defined as missing in more than 3% of samples.

### Differential gene expression analysis

**Fig. 1:**
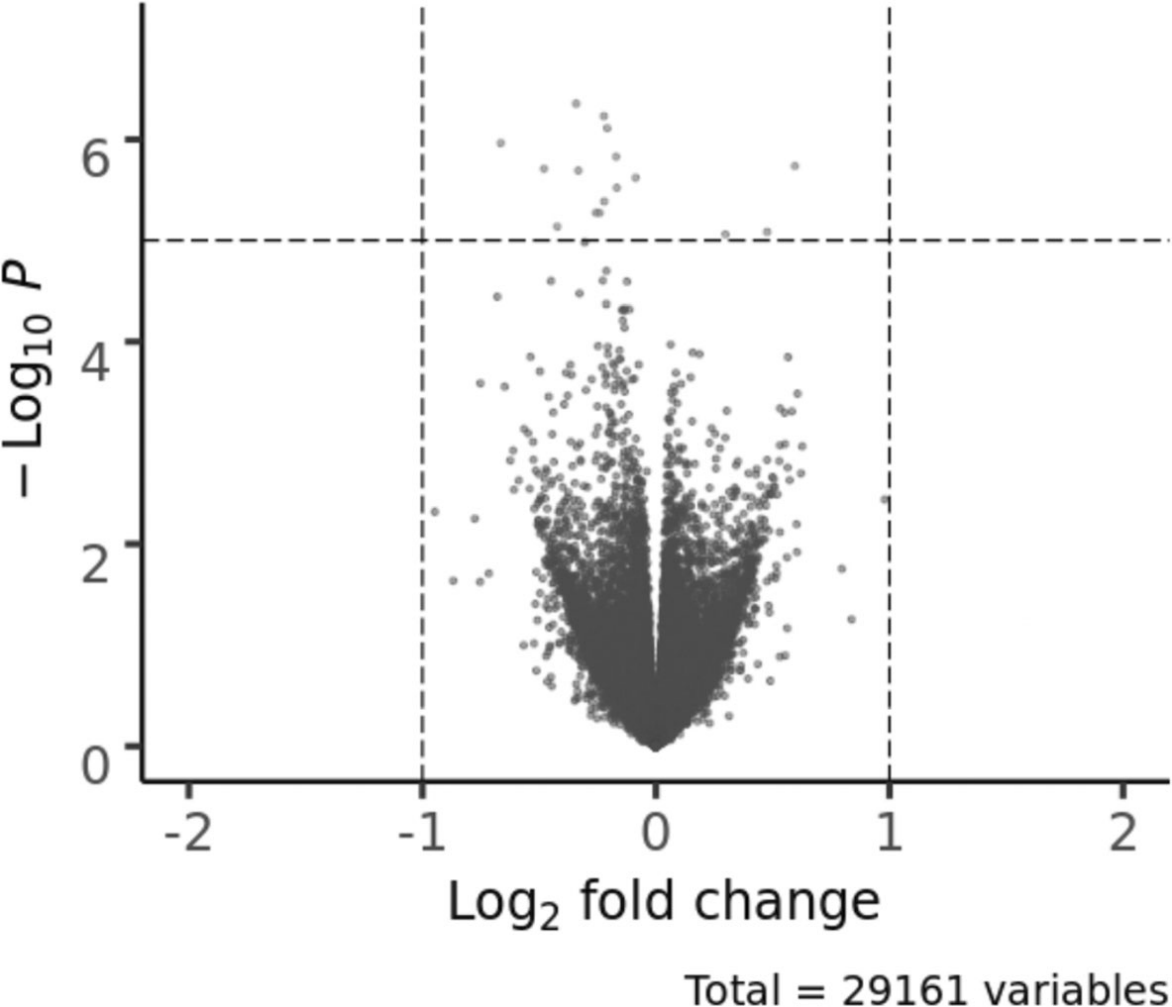
Volcano plot of differential gene expression of cachectic vs. non-cachectic COPD patients from COPDGene. The plot shows the log2 fold change on the x-axis and the unadjusted p-value on the y-axis (on the -log10 scale). Blue dots represent genes with a fold changed greater than or equal to + 1 and a *p*-value < 0.05

Differential gene expression analysis comparing cachectic and non-cachectic COPD patients from COPDGene was performed using the voom [19] and limma [20] R packages. Linear regression models were fit for each transcript and adjusted to control for age, gender, white blood cell (WBC) percentages, and significant SVs (*N* = 27). Significance for differentially expressed transcripts in COPDGene was defined as an FDR *p*-value < 0.05 adjusted for number of transcripts (Fig. 1).

In ECLIPSE*,* only probes mapping to a gene that was significantly differentially expressed in COPDGene were evaluated using linear regression models. A *P* < 0.05 was considered to be a significant replication in ECLIPSE. Models were adjusted to control for age, gender, and WBC percentages. In instances were multiple probes mapped to a single gene, the probe with the highest mean expression value was selected [21].

Gene overlap enrichment between genes significantly differentially expressed in COPDGene and ECLIPSE were calculated using the GeneOverlap R package [22] using a two-tailed Fisher’s exact to calculate the FDR corrected *p*-value between overlapping genes.

### Pathway analysis

Gene set enrichment analysis (GSEA) of genes significantly differentially expressed in COPDGene and for those that replicated in ECLIPSE was performed using the Molecular Signatures Database (MSigDB) v6.1 [23]. This included gene set collections comprised of the hallmark gene set (*N* = 50 gene sets), curated gene sets (*N* = 4762 gene sets), Gene Ontology (GO) gene sets (*N* = 5917 gene sets), oncogenic signatures (*N* = 189 gene sets), and immunogenic signatures (*N* = 4872 gene sets). GSEA significance was defined as having an FDR-q *p*-value less than 0.05.

## Results

### Demographics

The prevalence of cachexia was 13.7% in COPDGene participants with COPD (Table 1).

**Table 1:** COPDGene Sample Demographics. Descriptive values for COPD patients (*N* = 400) in COPDGene with gene expression data by Cachectic (*N* = 55) and Non-Cachectic (*N* = 345) co-morbid status. Unless otherwise noted values denote mean (SD)

Demographic	Cachectic	Non-Cachectic	***P***-value
**N (%)**	55 (13.7)	345 (86.3)	
**Age**	68.1 + 9.2	68.8 + 8.1	0.52
**Sex** N(%)			0.049
Male	26 (47.3)	215 (62.3)	
Female	29 (52.7)	130 (37.7)	
**FEV1pp** (%)	44.0 + 18.1	54.0 + 18.1	< 0.001
**Pack Years Smoking**	46.8 + 19.2	53.5 + 26.0	0.027
**BMI** (kg/m^2^)	22.0 + 4.9	28.8 + 6.0	< 0.001

FEV1pp – Forced Expiratory Volume in One Second Percent Predicted, BMI - Body Mass Index, SD – Standard Deviation

There was no significant difference between the cachectic and non-cachectic COPD participants in COPDGene in terms of age (Table 1). On average, cachectic COPD subjects were more likely to be female (52.7% vs. 37.7%, *p* = 0.049), have lower lung function (FEV_1_% predicted 44.0 + 18.1 vs. 54.0 + 18.1, *p* < 0.001), and had less intensive smoking histories (46.8 + 19.2 vs. 53.5 + 26.0 pack years, *p* = 0.027) (Table 1). Cachectic COPD participants had lower BMI compared to non-cachectic COPD participants (22.0 + 4.9 vs 28.8 + 6.0, p < 0.001) (Table 1). As replication analyses were performed using data from the ECLIPSE study, we compared the characteristics of COPD participants included in the analyses in both studies in terms of age, sex, smoking duration, lung function, and BMI (Table 2). There were no significant differences between participants in COPDGene and ECLIPSE in terms of cumulative smoking exposure, lung function (FEV1 percent predicted), and BMI (Table 2). COPD participants in ECLIPSE were slightly younger (64.9 ± 5.3 vs. 68.7 ± 8.2 years, *p* < 0.05).

**Table 2:** Sample Demographics. Descriptive values for COPDGene and ECLIPSE subjects with COPD and gene expression data. Unless otherwise noted values denote mean + (SD)

Demographic	COPDGene Study	ECLIPSE Study	P-value
**N**	400	114	
**Age**	68.7 + 8.2	64.9 + 5.3	p < 0.05
**Sex** N(%)			p < 0.05
Male	241(60.3)	78 (68.4)	
Female	159 (39.7)	36 (31.6)	
**FEV1pp** (%)	52.7 + 18.4	50.5 + 15.1	0.20
**Pack Years Smoking**	52.6 + 25.3	47.6 + 28.3	0.08
**BMI** (kg/m^2^)	27.9 + 6.3	27.0 + 5.5	0.11

FEV1pp – Forced Expiratory Volume in One Second Percent Predicted, BMI - Body Mass Index, SD – Standard Deviation

### Differential gene expression in COPDGene

In COPDGene, 23 transcripts were significantly differentially expressed between cachectic and non-cachectic COPD participants (Table 2). Of these genes, 22 were downregulated among those with COPD cachexia.

### Replication in ECLIPSE

In ECLIPSE, only 14 of the 23 significantly differentially expressed genes in COPDGene were measured on the Affymetrix expression array. All of which were also significantly differentially expressed among participants with COPD cachexia in ECLIPSE (Table 3). These genes include *ALAS2, ANK1, ASCC2, CDC34, GUCD1, PLEK2, TNS1, SPTB, TRIM58, RILP, PPP2R5B, SMIM24, SLC25A39,* and *UBXN6* (Table 3)*.* In addition, gene set overlap enrichment analysis of significantly differentially expressed genes in COPDGene and ECLIPSE identified these 14 genes as having a significant overlap (FDR-*p* = 7.9 × 10^− 11^).

**Table 3:** Genes significantly differentially expressed between cachectic^1^ and non-cachectic COPD

Gene	Name	COPDGene		ECLIPSE^3^	
		Log FC	FDR-value^**2**^	Log FC	FDR-value^**2**^
*ALAS2*	5′-Aminolevulinate Synthase ^2^	− 0.42	0.015	−0.98	0.00010
*ANK1*	Ankyrin 1, Erythrocytic	−0.23	0.036	−0.80	0.0060
*ASCC2*	Activating Signal Cointegrator 1 Complex Subunit ^2^	−0.21	0.0074	−0.53	0.012
*BACH2*	BTB Domain and CNC Homolog ^2^	−0.17	0.0074	–	–
*CDC34*	Cell Division Cycle 34	−0.22	0.011	−0.58	0.0066
*CSF2RB*	Colony Stimulating Factor 2 Receptor Beta Common Subunit	−0.12	0.036	–	–
ENSG00000231393	Novel Transcript	−0.45	0.036	–	–
ENSG00000236283	Novel Transcript	−0.68	0.046	–	–
*GUCD1*	Guanylyl Cyclase Domain Containing ^1^	−0.17	0.0088	−0.36	0.0060
*IGHG1*	Immunoglobulin Heavy Constant Gamma ^1^	0.59	0.0074	–	–
*MIR186*	MicroRNA 186	−0.48	0.0074	–	–
*PLEK2*	Pleckstrin ^2^	−0.33	0.044	− 1.04	0.0027
*PPP2R5B*	Protein Phosphatase 2 Regulatory Subunit B’Beta	−0.26	0.012	−0.30	0.012
*RILP*	Rab Interacting Lysosomal Protein	−0.24	0.012	−0.51	0.012
*RNF149*	Ring Finger Protein 149	−0.087	0.0078	–	–
*SLC25A39*	Solute Carrier Family 25 Member 39	−0.22	0.0074	−0.57	0.0053
*SMIM24*	Small Integral Membrane Protein ^2^	−0.66	0.0074	−0.71	0.025
*SPTB*	Spectrin Beta, Erythrocytic	−0.33	0.0074	−0.77	0.0060
*TICAM1*	Toll Like Receptor Adaptor Molecule ^1^	0.48	0.016	–	–
*TNS1*	Tensin ^1^	−0.31	0.018	−1.07	0.0010
*TRIM58*	Tripartite Motif Containing 58	−0.34	0.0074	−0.90	0.0036
*TRPM2*	Transient Receptor Potential Cation Channel Subfamily M Member ^2^	0.30	0.016	–	–
*UBXN6*	UBX Domain Protein 6	−0.21	0.032	−0.55	0.012

*FC* Fold Change

^1^In COPDGene, cachexia was defined at visit two, approximately five years from baseline, as either unintentional weight loss greater than 5% in the past year or low BMI in addition to one of three criteria: low 6MWD, anemia or low FFMI. Unintentional weight loss was calculated at visit two using self-reported unintentional weight loss in the past year. Low 6MWD and anemia were classified using visit two data. FFMI was derived from pectoralis muscle area on baseline chest computed tomography scans [18]. In ECLIPSE, cachexia was defined as weight loss greater than 5 % in the past 12 months or low BMI and at least three of the five criteria: low six-minute walking distance, anorexia, abnormal biochemistry (anemia or high CRP), fatigue, and low FFMI

^2^In COPDGene, FDR was calculated based on 65,988 transcripts. In ECLIPSE, FDR was calculated based on *N* = 14 significantly differently expressed probes present in COPDGene

^3^Missing values were not significantly replicated in ECLIPSE

### Pathway analysis

The 23 differentially expressed genes associated with COPD cachexia in the discovery cohort were significantly enriched for gene sets involved in heme metabolism (Hallmark Heme Metabolism, FDR *p*-value = 5.92 × 10^− 7^) and heme biosynthesis (GO Heme Biosynthetic Process, FDR p-value = 3.85 × 10^− 2^), among others (Table S2). The 14 significantly differentially expressed COPD cachexia genes in the discovery which replicated were enriched with genes involved in heme metabolism (Hallmark Heme Metabolism, FDR-*p* = 4.50 × 10^− 8^; *ALAS2, ANK1, TNS1, SPTB, TRIM58, PPP2R5)* and heme biosynthesis (GO Heme Biosynthetic Process, FDR-*p* = 2.45 × 10^− 2^; *ALAS2, SLC25A39*), among others (Table 4). *ASCC2, CDC34, UBXN6, PLEK2, RILP, SMIM24* and *GUCD1* were not enriched in any significant pathways.

**Table 4:** Gene Set Enrichment Analysis of Genes Associated with COPD Cachexia in COPDGene which replicated in ECLIPSE

Gene Set Name	N Genes in Set	N Genes Overlap	FDR p-value
GSE34205 RSV vs Flu INF Infant PBMC Up	200	7	3.27 × 10^−10^
Hallmark Heme Metabolism	200	6	4.50 × 10^−8^
Valk AML Cluster 7	28	3	0.00023
GSE34205 Healthy vs RSV INF Infant PBMC Down	200	4	0.00067
Chyla CBFA2T3 Targets Down	242	4	0.0011
Steiner Erythrocyte Membrane Genes	15	2	0.017
GO Heme Biosynthetic Process	20	2	0.025
Welch GATA1 Targets	22	2	0.025
PRC2 SUZ12 Up V1 Up	191	3	0.025
REACTOME Interaction Between L1 AND Ankyrins	23	2	0.025
GSE16522 Anti CD3CD28 Stim VS Unstim Memory CD8 T-Cell Up	199	3	0.025
Valk AML Cluster 8	26	2	0.025
GO Tetrapyrrole Biosynthetic Process	27	2	0.025
GO Heme Metabolic Process	29	2	0.029
GO Porphyrin Containing Compound Metabolic Process	36	2	0.041
Hofmann Cell Lymphoma Down	39	2	0.046

## Discussion

**Fig. 2:**
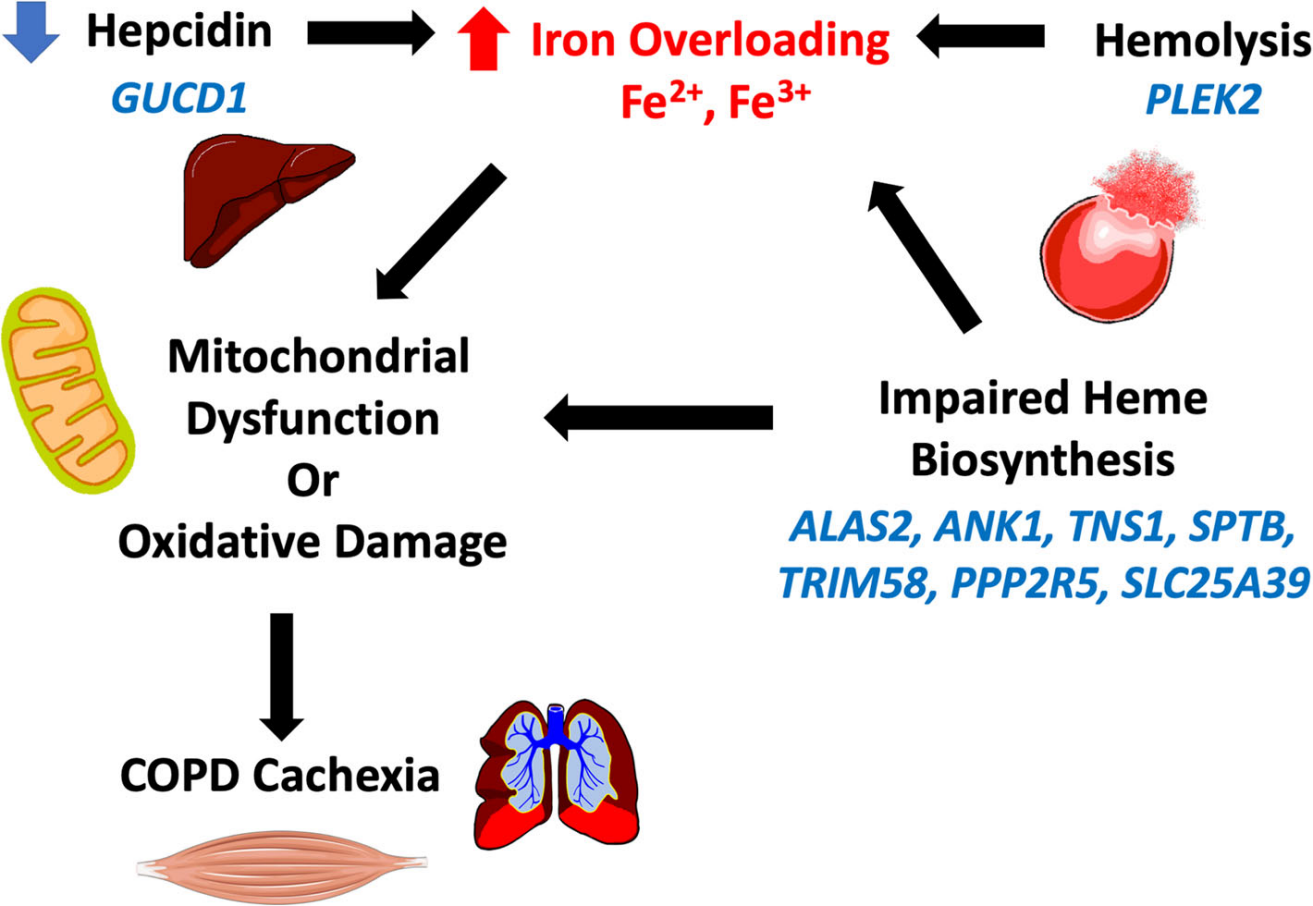
Summary of key findings in relationship to a putative role of iron in COPD cachexia. Genes in blue are significantly downregulated in COPD cachexia in manuscript analyses

In this study, we identified novel gene expression signatures associated with COPD cachexia in two independent cohorts of participants with moderate to severe COPD. All significant genes were downregulated among cachectic COPD patients. Several of these genes are involved in the regulation of heme metabolism (*ALAS2, ANK1, TNS1, SPTB, TRIM58, PPP2R5)* and biosynthesis (*ALAS2* and *SLC25A39*). These findings may have implications for cachexia disease surveillance and drug development. Impaired heme metabolism and biosynthesis, decreased hepcidin production, and hemolysis may contribute to COPD cachexia through the induction of iron overloading leading to oxidative tissue damage, mitochondrial dysfunction, and aberrant repair (Fig. 2).

Disruption of iron homeostasis in lung disease overall has received attention as a potential pathobiological mechanism in COPD [24–26]. Recently, Sato et al. [26] reported mice fed an iron deficient diet were more susceptible to cigarette smoke developing severe emphysema and lung hyperinflation. This finding conflicts with an earlier report by Cloonan et al. [25] where mice fed an iron deficient diet or given an iron chelator were less susceptible to cigarette smoke exposure. In keeping with Cloonan et al. [25], our findings may point to a role for iron toxicity through impaired heme biosynthesis and metabolism as a number of genes involved with these processes were downregulated in COPD cachexia (Fig. 2). Heme is an essential component of hemoglobin and anemia is a component of the criteria used to define cachexia. Anemia is a common co-morbidity in COPD [24]. We performed a sensitivity analysis to evaluate whether the 23 genes significantly differentially expressed among subjects with COPD cachexia were also associated with anemia and found no significant association (data not shown). A genome-wide expression study of skeletal muscle biopsies from upper gastro-intestinal cancer patients highlighted *ALAS1* as downregulated in patients with cancer cachexia [27]. *ALAS2* and *ALAS1* encode the same enzyme, 5′-Aminolevuilnnate Synthetase, which plays a key role in heme synthesis [28]. Previous studies have demonstrated that *ALAS1* and *ALAS2* expression levels are increased during erythropoiesis in healthy individuals [28]. *ALAS2* is erythroid-specific whereas *ALAS1* is expressed in many tissues, including skeletal muscle. This may indicate defective heme biochemistry has ramifications outside of circulating red blood cells which could be relevant to both cancer and COPD cachexia. *SLC25A39* encodes the solute carrier family 25 member 39, a mitochondrial membrane protein which plays a role in iron homeostasis and metabolism [29]. Specifically, *SLC25A39* is involved in iron transportation and transfer, and interruptions in *SLC25A39* biogenic activity have been shown to result in iron overloading [29]. *ANK1* (encodes ankyrin 1) and *SPTB* (encodes the erythrocytic protein spectrin beta) are involved in erythrocyte membrane stability. Mutations in *ANK1* and *SPTB* have been linked to erythrocyte membrane disorders including hereditary spherocytosis, in which clinically significant iron overload is commonly seen [30]. *PPP2R5B (*protein phosphatase 2 regulatory subunit B’beta) and *TRIM58* (tripartite motif containing 58) function as regulators of erythropoiesis and mutations in these genes can lead to defective erythropoiesis and [31, 32] iron overloading [33]. *TNS1* (Tensin 1) plays a role in heme metabolism and functions in the formation of fibrillar adhesion in muscle. Previous studies have identified an association between genetic variants in *TNS1* and elevated risk of COPD and lung function decline [34]. Transcriptomic analysis has identified *TNS1* as downregulated and differentially expressed in hypoxic alveolar cells [35]. Lung parenchymal destruction in COPD makes it difficult for the body to take in sufficient oxygen and can lead to hypoxemia (low blood oxygen saturation). Hypoxemic COPD patients often present with polycythemia, which suggests increased production of erythrocytes may be one mechanism for the body to maximize delivery of oxygen from the lungs [36]. Downregulation of *TNS1* and other genes playing a critical role in heme metabolism could inhibit the body’s ability to effectively sequester the elevated levels of iron produced leading to an excess of iron.

In our study, *PLEK2* was significantly downregulated in cachectic COPD patients. *PLEK2* encodes pleckstrin-2, a protein playing a critical role in the protection of erythroblasts from oxidative damage [37]. Specifically, *PLEK2* functions in the structural organization of the erythroblast to prevent the localization of cofilin (an actin-binding protein) to the mitochondria where it induces apoptosis [28, 37]. Previous studies have demonstrated pleckstrin-2 knockout results in erythrocyte apoptosis and inhibition of differentiation, a precursor to cell-free iron loading [37]. Our study indicates downregulation of *PLEK2* could result in increased rates of hemolysis resulting in iron overloading (Fig. 2).

Hepcidin is essential for sequestering iron in the body and downregulation of hepcidin has been shown to cause severe iron overloading [38]. One gene, *GUCD1*, was downregulated in cachectic COPD patients. *GUCD1* (guanylyl cyclase domain containing 1) is a ubiquitous protein that plays a role in regulating the growth of liver cells [39]. Hepcidin, produced in liver cells, degrades ferroprotein, which is responsible for releasing iron from cells [38]. Downregulation of *GUCD1* in our study may suggest one mechanism of iron-overloading due to impaired hepcidin production (Fig. 2).

Iron overload due to inhibited iron metabolism has been implicated in increased production of reactive oxygen species, mitochondrial dysfunction, and oxidative stress [24]. Interestingly, mitochondrial dysfunction is one mechanism thought to contribute to the development of muscle wasting and dysfunction in COPD via buildup of oxidative stress [2]. In our study, the downregulation of genes involved in heme metabolism and biosynthesis, hepcidin production, and erythrocyte membrane stability may contribute to iron-overloading. Iron overloading has been implicated in the generation of mitochondrial dysfunction and oxidative damage, which are hallmarks of COPD cachexia pathogenesis (Fig. 2) [40].

Our study has several strengths and limitations. A strength of our study is that it presents the largest genome-wide expression study of cachexia in patients with COPD. We took advantage of a genome-wide approach which highlighted new candidate genes associated with COPD cachexia such as those involved with heme biochemistry. Another strength of our study was our ability to phenotype cachexia in our replication cohort (ECLIPSE) according to the criteria from the cachexia consensus definition [12], whereas previous studies have focused on COPD cachexia-associated proxy traits such as low FFMI or low BMI that lack specificity. Finally, we were also able to replicate 14 genes as a potential markers of COPD cachexia in an independent cohort of participants with COPD. Among the limitations, we were not able to test whether all 23 significantly differentially regulated genes in COPDGene replicated in ECLIPSE due to limitations with the microarray not having probes for all 23 genes. It is possible some of these genes would also replicate but requires further study in a COPD cohort with RNA-sequencing data. There was also limited phenotype data available to characterize cachexia in COPDGene to code the 5 criteria of the consensus definition; thus, we used the maximal set available. As not all criteria were available to categorize cachexia using the Evans consensus definition, participants with COPD may have been miscategorized. However, the criteria we used to classify cachexia in ECLIPSE were much more stringent and we replicated 14/23 findings. Further, unintentional weight loss in COPDGene was self-reported, which can be unreliable. We also dichotomized 6MWD (6MWD < 350 m) as a surrogate for muscle strength which could have misclassified some participants. Although it is reasonable to infer participants with very low 6MWD have low muscle strength, other factors such as cardiac and pulmonary function may affect 6MWD. We also analyzed gene expression data generated at a single time point. A longitudinal analysis would have provided a deeper and more powerful understanding of the changes in gene expression over time. Future analyses could prove useful in understanding the changes that occur over time in cachectic COPD patients and even prior to the development of cachexia to define at-risk populations. Also, we did not use qRT-PCR to validate significant findings from COPDGene However, our discovery analyses were performed with a fairly large sample size of 400 COPD subjects with replication in an independent cohort of COPD subjects. Finally, peripheral blood was used to evaluate gene expression in cachectic COPD subjects as opposed to skeletal muscle. However, our study replicates abovementioned findings using skeletal muscle biopsies to evaluate gene expression in patients with cancer cachexia [27]. Additionally, a peripheral blood sample is less invasive than a skeletal muscle biopsy, an important factor to consider due to the advanced age and accompanying frailty in COPD patients [1].

## Conclusions

In conclusion, this study demonstrates the utility of genome-wide expression analyses to identify novel molecular genes and pathways associated with COPD cachexia. Specifically, our results indicated genes involved in heme biosynthesis and metabolism were significantly downregulated in cachectic patients with COPD. These results highlight impaired heme metabolism and biosynthesis as potential mechanisms for COPD cachexia pathogenesis via free iron build up, oxidative tissue damage, and aberrant repair. Further studies are warranted to determine potential efficacy of therapeutic targeting to prevent, attenuate, or even possibly reverse COPD cachexia.

## Supplementary information

**Additional file 1: Table S1.** ECLIPSE Sample Demographics. Descriptive values for COPD patients (*N* = 114) in ECLIPSE with gene expression data by Cachectic (*N* = 9) and Non-Cachectic (*N* = 105) co-morbid status. Unless otherwise noted values denote mean (SD). **Table S2.** Gene set enrichment analysis of 23 significantly (FDR *p*-value < 0.05) differentially expressed between cachectic and non-cachectic COPD patients in COPDGene.

**Additional file 2: Figure S1.** Principle component analysis on SV residualized expression data of (*N* = 400) COPDGene subjects with COPD. Principal component 1 (PC1) is presented on the x-axis and the second principle component on the y-axis (PC2) after adjustment for significant surrogate variables (*N* = 27). Numbers in parentheses denotes the proportion of variance captured by each component. Red dots indicate COPD subjects with cachexia. Blue dots indicate COPD subjects without cachexia.

## Data Availability

The datasets generated and/or analyzed during the current study for are available from dbGaP and GEO on reasonable request. [https://www.ncbi.nlm.nih.gov/geo/, https://www.ncbi.nlm.nih.gov/gap/].
