# Supplementary material for: Heme metabolism genes Downregulated in COPD Cachexia
Source: Respir Res. 2020 May 1;21:100. doi: 10.1186/s12931-020-01336-w (PMC7193359; doi:10.1186/s12931-020-01336-w)
Supplement: Supplementary file 1 — Additional file 1: Table S1. ECLIPSE Sample Demographics. Descriptive values for COPD patients (N = 114) in ECLIPSE with gene expression data by Cachectic (N = 9) and Non-Cachectic (N = 105) co-morbid status. Unless otherwise noted values denote mean (SD). Table S2. Gene set enrichment analysis of 23 significantly (FDR p-value < 0.05) differentially expressed between cachectic and non-cachectic COPD patients in COPDGene. [file 12931_2020_1336_MOESM1_ESM.pdf]

Table S1: ECLIPSE Sample Demographics. Descriptive values for COPD patients (N = 114) in ECLIPSE with gene expression data by Cachectic (N = 9) and Non-Cachectic (N = 105) co-morbid status. Unless otherwise noted values denote mean (SD).

Demographic	Cachectic	Non-Cachectic	P-value
**N (%)**	9 (7.9)	105 (92.1)	
**Age**	64.2±5.4	64.9±5.4	0.69
**Sex** N (%)			0.62
Male	5 (55.6)	73 (69.5)	
Female	4 (44.4)	36(31.6)	
**FEV1pp** (%)	39.9±17.6	51.4±14.7	0.028
**Pack Years Smoking**	44.0±15.4	48.0±29.2	0.69
**BMI** (kg/m^2)	21.1±4.8	27.6±5.3	<0.001

FEV1pp – Forced Expiratory Volume in One Second Percent Predicted, BMI - Body Mass Index, SD – Standard Deviation

Table S2: Gene set enrichment analysis of significantly (FDR p-value < 0.05) differentially expressed between cachectic and non-cachectic COPD patients in COPDGene.

Gene Set Name	N Genes in Set	N Genes Overlap	P-value	FDR P-value
GSE34205 RSV vs Flu INF Infant PBMC Up	200	7	4.99×10^{-13}	7.87×10^{-9}
Hallmark Heme Metabolism	200	6	7.49×10^{-11}	5.92×10^{-7}
Nuytten EZH2 Targets Up	1037	7	4.66×10^{-8}	2.45×10^{-4}
Valk AML Cluster 7	28	3	1.37×10^{-7}	5.41×10^{-4}
GSE34205 Healthy vs RSV INF Infant PBMC Down	200	4	7.92×10^{-7}	0.00250
CHYLA CBFA2T3 Targets Down	242	4	1.69×10^{-6}	0.00445
McBryan Terminal End Bud Up	12	2	8.48×10^{-6}	0.0191
Steiner Erythrocyte Membrane Genes	15	2	1.35×10^{-5}	0.0266
REACTOME Immune System	933	5	1.72×10^{-5}	0.0302
GO Heme Biosynthetic Process	20	2	2.44×10^{-5}	0.0385
Welch GATA1 Targets	22	2	2.96×10^{-5}	0.0417
REACTOME Interaction Between L1 And Ankyrins	23	2	3.24×10^{-5}	0.0417
Valk AML Cluster 8	26	2	4.16×10^{-5}	0.0417
GO Tetrapyrrole Biosynthetic Process	27	2	4.50×10^{-5}	0.0417
PRC2 SUZ12 UP V1 Up	191	3	4.60×10^{-5}	0.0417
GO Heme Metabolic Process	29	2	5.20×10^{-5}	0.0417
GSE16522 ANTI CD3CD28 Stim vs Unstim Memory CD8 T-cell Up	199	3	5.20×10^{-5}	0.0417
GSE19401 PAM2CSK4 VS Retinoic Acid Stim Follicular DC Up	200	3	5.28×10^{-5}	0.0417
GSE24292 WT vs PPARG KO Macrophage Up	200	3	5.28×10^{-5}	0.0417
GSE38304 MYC neg vs Pos GC B-cell Up	200	3	5.28×10^{-5}	0.0417

FDR - False Discovery Rate. Gene set collections from MSigDB include H, C2, C5, C6, and C7.
