# Supplementary figures and images for: Heme metabolism genes Downregulated in COPD Cachexia
Source: Respir Res. 2020 May 1;21:100. doi: 10.1186/s12931-020-01336-w (PMC7193359; doi:10.1186/s12931-020-01336-w)

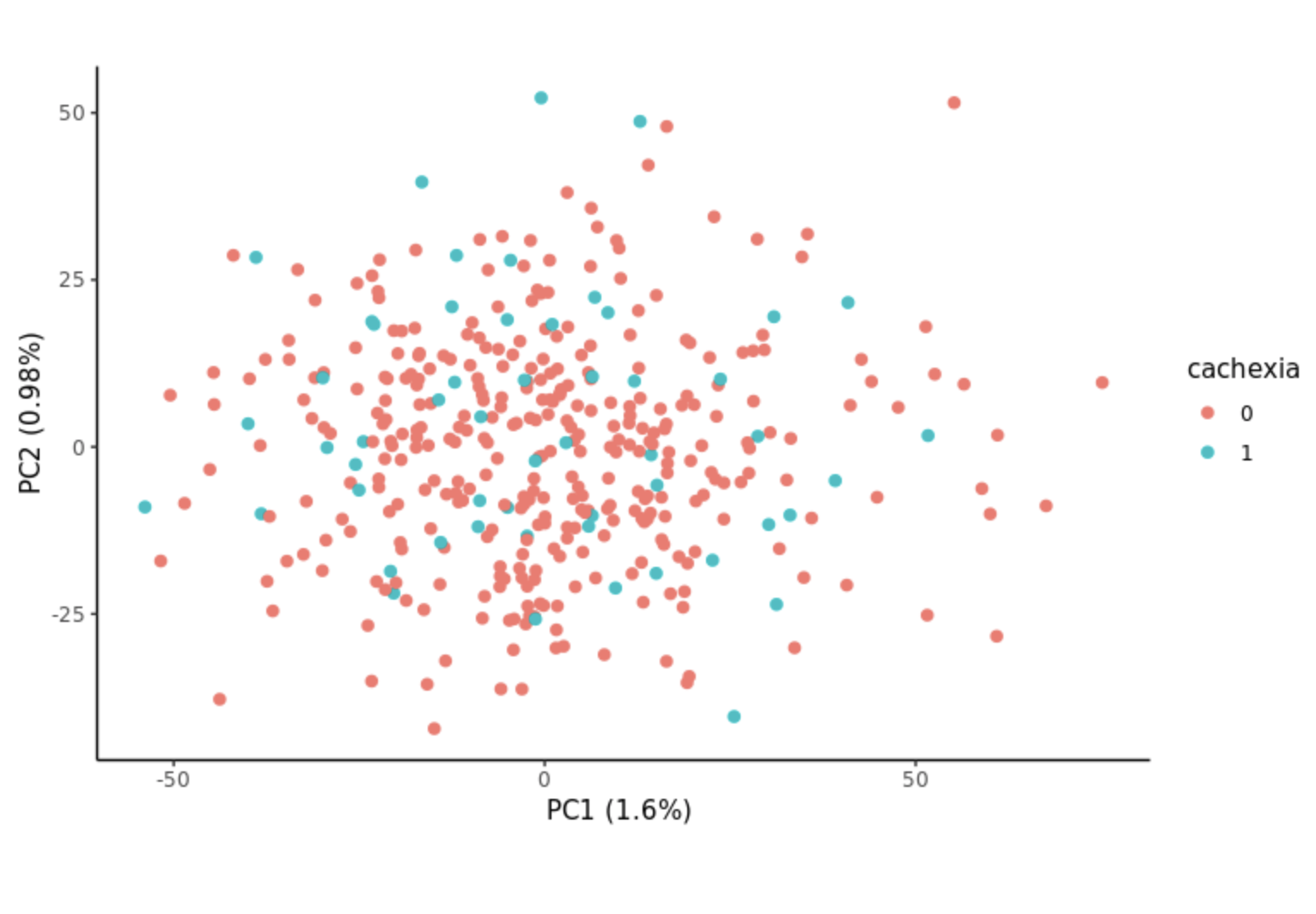

Supplement: Supplementary file 2 — Additional file 2: Figure S1. Principle component analysis on SV residualized expression data of (N = 400) COPDGene subjects with COPD. Principal component 1 (PC1) is presented on the x-axis and the second principle component on the y-axis (PC2) after adjustment for significant surrogate variables (N = 27). Numbers in parentheses denotes the proportion of variance captured by each component. Red dots indicate COPD subjects with cachexia. Blue dots indicate COPD subjects without cachexia. [file 12931_2020_1336_MOESM2_ESM.png]
